# Multi-Service Multiplexing System Based on Visible Light Communication

**DOI:** 10.3390/s25237207

**Published:** 2025-11-26

**Authors:** Yangyu Zhang

**Affiliations:** 1 State Key Laboratory of Optoelectronic Materials and Devices, Institute of Semiconductors, Chinese Academy of Sciences, Beijing 100083, China; zhangyangyu22@mails.ucas.ac.cn; Tel.: +86-159-7358-6152; 2 College of Materials Science and Opto-Electronic Technology, University of Chinese Academy of Sciences, Beijing 100049, China

**Keywords:** visible light communication, time division multiplexing, signal processing, audio communications

## Abstract

As the Internet of Things (IoT) and communication technologies continue to evolve, the value of multi-service multiplexing in visible light communication (VLC) systems has been increasingly recognized, particularly in addressing the scarcity of wireless spectrum resources. This study reconstructed the stereo transmission protocol through methods such as dynamic level control, designed a timer interrupt service routine with a double buffer, and reassigned channel status bits in the frame processing function. Consequently, a multi-service multiplexing system based on VLC was designed and implemented. The system enables hybrid transmission of audio signals (1–21.6 kHz) and character data (300–1200 bps) via a single channel, accurately reproducing both voice and text input over a 3.2 m communication range. The system, benefiting from the directional nature of visible light communication, exhibits inherent robustness to multipath-induced interference in dominant line-of-sight (LoS) scenarios and can be easily integrated into existing lighting networks. Featuring a simple architecture and cost-effective design, this solution shows promise for deployment in RF-sensitive areas requiring multi-service communication.

## 1. Introduction

Radio frequency communication technology, particularly Radio Frequency Identification (RFID), has been widely adopted across various sectors, enhancing daily operations and convenience in areas such as transportation, retail, library systems, and smart homes. However, there remain specialized areas where traditional radio frequency communication is not the optimal solution. For instance, intensive care units (ICUs), nuclear power plants, and underground mines, among others. These areas are collectively known as radio frequency-sensitive zones. VLC technology serves as the preferred communication solution for these areas [1]. VLC utilizes the visible light spectrum, with wavelengths ranging from 400 to 760 nm, for communication effectively circumvents the generation of radio frequency interference [2]. Due to the inherent characteristics of visible light, such as its visibility and linear propagation, VLC technology inherently possesses a high level of confidentiality, making it suitable for applications in environments where security is paramount. Given its high security, good confidentiality, and low power consumption, VLC stands out as a promising new communication technology, particularly suited for special scenarios such as indoor navigation, smart home applications, and intelligent transportation systems.

The widespread adoption of technologies like 5/6G, the industrial Internet, and the IoT has introduced new challenges for communication networks [3]. A single network may need to handle multiple services simultaneously, such as voice, video, AR/VR, industrial control, and large-scale sensor data. Research into hybrid transmission methods for multiple services can enhance the spectral utilization rate and improve the efficiency of network resources. In recent years, multi-service multiplexing and high-performance transmission technologies in visible light communication have become a research hotspot, aiming to enhance spectral efficiency, system capacity, and network intelligence. Current research primarily focuses on the following directions:

To improve physical layer transmission performance, pre-coding and advanced modulation techniques have been extensively studied to address inherent challenges in VLC systems. For instance, Jia Kejun et al. [4] theoretically analyzed and simulated the effectiveness of pre-coding techniques (such as DFT, DCT, and DHT) in Optical Orthogonal Frequency Division Multiplexing (O-OFDM) systems. The study demonstrated that under multipath channel conditions, pre-coding can effectively mitigate frequency-selective fading, homogenize the signal-to-noise ratio across subcarriers, thereby significantly improving system bit error rate performance while simultaneously suppressing the peak-to-average power ratio.

In MIMO-VLC systems with multi-user access, resource allocation strategies are critical. Chen Yong et al. [5] proposed an optimized solution for joint access point (AP) and power allocation in indoor scenarios with non-uniform user distribution. The study introduced an AP allocation algorithm based on channel gain weighting backtracking to balance network load, while improving the power allocation algorithm to ensure user service quality. This approach ultimately achieved improvements in both system throughput and user satisfaction.

In the pursuit of extreme data rates, research tends to adopt Wavelength Division Multiplexing (WDM) and more sophisticated modulation access techniques. Gunawan et al. [6] utilized a combination of RGB laser diodes, OFDM, and non-orthogonal multiple access technology to achieve a combined data rate of up to 21.01 Gbit/s, while implementing spatial multiplexing for multiple users through a fast-switching mirror. Chen et al. [7] proposed a space–wave–time-division multiple access scheme based on tri-color LEDs, achieving 750 Mbps transmission with a zero-bit error rate, which significantly enhanced the user capacity of two-way VLC networks.

With the advancement of IoT and sustainable development, research has shifted focus to heterogeneous network integration and energy efficiency. Tang et al. [8] developed a hybrid VLC/RF system where the VLC downlink simultaneously transmits data and harvests energy, which powers IoT devices for RF uplink communication, establishing an energy-efficient communication framework.

However, we observe that most of the aforementioned studies focus on enhancing parallel transmission performance between base stations and multiple users or pursuing extreme speeds in the Gbps range. These systems typically rely on high-performance optoelectronic devices, complex digital signal processors, or precision optical components, resulting in high system costs and complexity that make them difficult to deploy simply in low-cost, single-user multi-service scenarios. In contrast, this study takes a different approach by addressing the concurrent communication needs of a single user (especially fixed users in RF-sensitive areas) for multiple heterogeneous services (such as audio and data). In this research direction, recent research includes the following: Dong et al. developed a dual-channel VLC system, which successfully integrated video and voice hybrid transmission capabilities, achieving a communication distance of 15 cm [9]. Tian et al. devised a VLC system that can unidirectionally transmit voice signals and monitor temperature, achieving a transmission distance of up to 2.5 m [10]. The core innovation of this paper lies in proposing a highly cost-effective software–hardware collaborative design:

Single-channel transmission of multiple services: By reconstructing a stereo audio transmission protocol, it achieves mixed transmission of audio and character data within a single illumination optical channel, suitable for the most common indoor lighting communication scenarios while avoiding crosstalk that may arise from multi-channel parallelism.

Breakthrough protocol reconfiguration: By dynamically controlling signal levels and redefining channel state bits, it cleverly associates transmission data types with channel states, breaking through the limitations of the traditional I2S protocol for non-audio data transmission and simplifying data processing.

A minimalist low-cost architecture: The entire system is controlled by two low-cost microcontrollers and a general-purpose audio code chip, without the need for a complex digital signal processor (DSP), FPGA, or precision optical design, resulting in a small system size, low cost, and strong practical application potential.

The time division multiplexing (TDM) technology demonstrates unique advantages in VLC systems. Firstly, TDM effectively mitigates multi-channel interference by allocating channels through time-division allocation, enabling all users to share the same frequency band. This approach prevents optical interference issues like color separation and wavelength filtering caused by multiple wavelengths or frequency bands, while simplifying hardware design for both transmitters and receivers. Secondly, in terms of system complexity and power consumption, TDM signals require only signal detection within designated time slots at the receiver end, featuring simple decoding algorithms that make it ideal for low-power terminals. Lastly, TDM exhibits high compatibility with VLC systems. Since light sources in VLC systems are typically synchronized with visible light signals (e.g., through LED flicker frequency), TDM’s time-division allocation can directly bind with the light source’s modulation clock, achieving high-precision synchronization. TDM can also be seamlessly integrated into existing communication protocols (such as IEEE802.15.7VLC standards [11]) without modifying the underlying physical layer design. Table 1 highlights TDM’s distinctive advantages over other multiplexing methods in VLC systems. In this paper, by using TDM technology, the stereo transmission protocol was redesigned, and an appropriate optical communication circuit was designed. The single-channel mixed transmission of audio and character is achieved, and an integrated transceiver design was made. The communication distance reached 3.2 m.

The structure of this paper is as follows: Section 2 introduces the overall architecture and clock design of the system; Section 3 details the redesign of the I2S transmission protocol; Section 4 discusses the design of the optical transceiver module and analyzes the link capacity; Section 5 presents the system test results, performance comparison analysis, and discusses the system limitations; Section 6 summarizes the paper and outlines future research directions.

## 2. System Architecture Design

**Figure 1:**
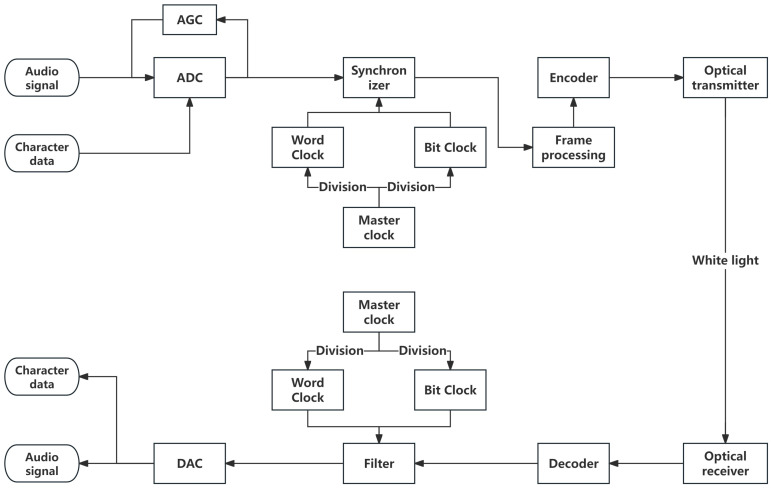
Block diagram of a visible light multiplexing communication system based on time-division multiplexing. AGC: Automatic Gain Control; ADC: Analog to Digital Converter; DAC: Digital to Analog Converter.

This research devised the structure of a dual-channel audio and character multiplexing system, as depicted in Figure 1, founded on the dual-channel audio communication system. Data transmission is partitioned into two channels, namely channel A and channel B, in accordance with time. Channel A transmits the audio signal obtained by collecting the external voice through the microphone, while channel B transmits the character data provided by external devices (sensors, computers, etc.). The character data and audio signal are then sent to the ADC together after gain control by the Automatic Gain Control (AGC). After clock synchronization and AES3 encoding, they are packaged into data frames and modulated onto the LED. The light emission module emits them in the form of white light. The light-receiving module converts the received light signal into an electrical signal and sends it to the next stage for decoding, desensitization, and digital-to-analog conversion in sequence. By reading at different time frames, respectively, the restored audio signal and character data can be obtained. The main design difficulties of the system include clock synchronization design and how digital signals can be detected and restored in the audio communication system. The following part of this section will explain the clock design of the system, and Section 3 will detail how to achieve the normal transmission of non-audio data in a standard audio communication system.

The key point of time-division multiplexing technology is how to precisely separate data frames by time. The audio communication of this system is designed according to the I2S Philips standard in the I2S transmission protocol, using the Word Clock (WCLK) and Bit Clock (BCLK) to match the data frames [12].

**Figure 2:**
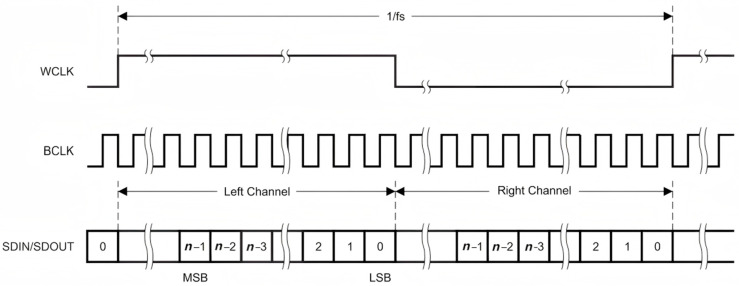
Data transmission timing design in multiplexed systems. WCLK: Word Clock; BCLK: Bit Clock; MSB: the Most Significant Bit; LSB: Least Significant Bit.

As shown in Figure 2, the highest bit (MSB) of the data in both the left and right channels is valid at the rising edge of the second BCLK after the WCLK change. Use WCLK to indicate the channel to which the data currently being sent belongs. When WCLK is 0, it indicates channel A, transmitting character data; when it is 1, it indicates channel B, transmitting audio signals. The WCLK signal becomes effective one clock before the highest bit of the current channel data. The WCLK signal varies at the falling edge of BCLK. The sender changes the data at the falling edge of the clock signal BCLK, and the receiver reads the data at the rising edge of the clock signal BCLK. The WCLK frequency is equal to the sampling frequency fs. One WCLK cycle (1/fs) includes sending data from both the left and right channels.

No matter how many valid bits of data there are, the highest bit of the data always appears at the WCLK change, that is, at the second BCLK pulse after the start of a frame. This enables the significant bit numbers at the receiving end and the sending end to be different. If the number of significant bits that the receiving end can handle is less than that of the sending end, it can abandon the excess low-bit data in the data frame. If the number of significant bits that the receiving end can handle is more than that of the sending end, it can make up for the remaining bits by itself. This synchronization mechanism makes the interconnection of devices more convenient and can effectively avoid data misalignment.

**Figure 3:**
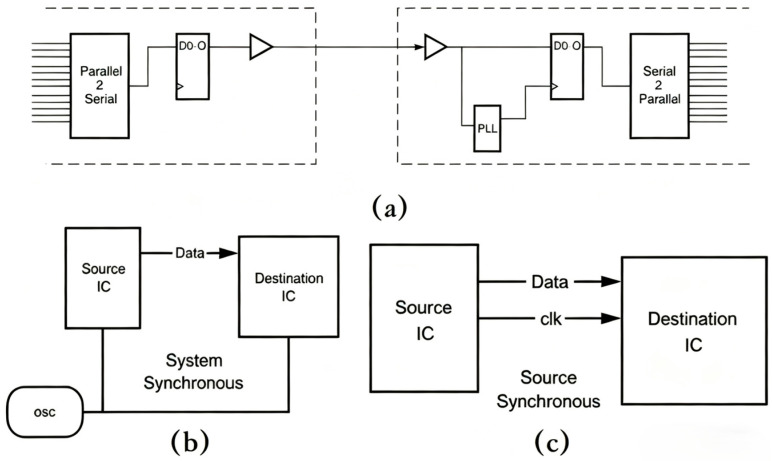
Three clock synchronization modes: (**a**) self-synchronization; (**b**) system synchronization; (**c**) source synchronization. PLL: Phase Locking Loop; OSC: Oscillator.

At present, there are three demultiplexing methods for time-division multiplexing, namely self-synchronization, system synchronization, and source synchronization. The principle block diagrams of each synchronization mode are shown in Figure 3. Self-synchronization is often used for communication between different chips, sending signals that include both the original data stream and the accompanying clock information. Its structure mainly includes serial/parallel signal conversion modules, parallel/serial signal conversion modules, and phase-locked loops. The structure is relatively complex and increases the amount of transmitted information. System synchronization and source synchronization are mainly used for internal communication within chips and are not very suitable for the mobile communication requirements in this study. This study designs and employs a time-decomposition multiplexing structure that does not require a phase-locked loop. The specific implementation method is that the clock matching at both the transmitting and receiving ends is provided by the master clock, and there is no need to transmit clock signals between the transmitting and receiving ends.

The typical auditory range of the human ear spans from approximately 20 Hz to 20 kHz, with the most sensitive frequencies lying between 1 kHz and 3 kHz. To accurately reconstruct an original signal from its samples, the sampling frequency must be at least twice the highest frequency present in the signal. This principle, also known as the Nyquist–Shannon sampling theorem,

(1) $$f_{s}\geq {2f}_{m}$$

According to calculations, the sampling rate ought to exceed 40 kHz. Considering the influence of quantization noise, a typical sampling rate of 48 KHz is more appropriate. The single-channel bit width is set to 24 bits. The parameter settings for data transmission are shown in Table 2.

## 3. Reconfiguration Design of the I2S Transmission Protocol

### 3.1. Dynamic Adjustment of Input Level

**Figure 4:**
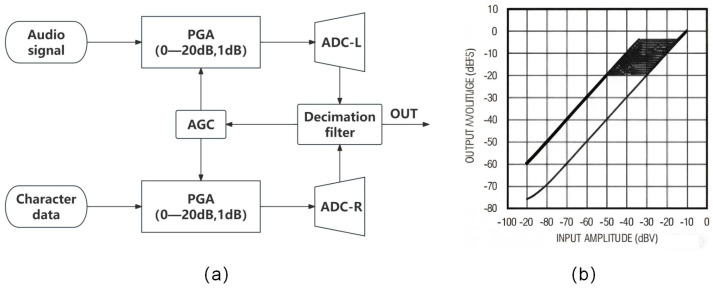
Automatic Gain Control (AGC) module structure and input–output characteristic curve: (**a**) AGC module structure block diagram; (**b**) AGC input–output characteristic curve. PGA: Programmable Gain Amplifier; ADC-L/R: Analog to Digital Converter-Left/Right.

Character data is a digital signal that must adhere to specific voltage amplitude standards in digital circuits. For instance, in TTL circuits, the standard output high level (VOH) is typically 2.4 V, while the standard output low level (VOL) is 0.4 V. Similarly, the minimum input high level (VIH) is 2.0 V, and the maximum input low level (VIL) is 0.8 V. After dividing by the input impedance of the system, the character data level is still much larger than the tiny current generated by the microphone to collect the audio signal and often appears too large. To address the uncertainties that may arise from such differences and ensure that the designed communication system has good audio performance under various conditions, in this research scheme, an AGC module was designed. Its structure and module effect are shown in Figure 4a. Its main function is to control the Programmable Gain Amplifier (PGA) based on the voltage collected at the output stage. The amplification capability of PGA is negatively feedback-regulated to keep the final sampling level output to the next stage constant within a range of 20 dB (Figure 4b), thereby expanding the dynamic range of signal levels that the system can handle.

### 3.2. Redefinition of Channel State Bits

**Figure 5:**
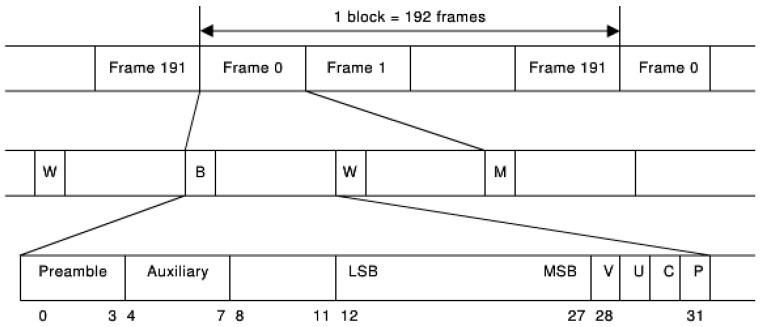
IEC60958 dual-channel data packet format. 0–3 bits: Preamble; 4–7 bits:Auxiliary Data; 8–27 bits:Audio Data; 28 bit: Validity Bit; 29 bit:User Bit; 30 bit: Channel Status Bit (C); 31 bit: Parity Bit.

The clock-synchronized mixed data is subsequently encoded using the AES3 method, which complies with the IEC60958 protocol. This process generates the dual-channel message data structure illustrated in Figure 5 [13]. The specific data format is as follows: A block is composed of 192 frames, and each frame stores a set of sample signals from two channels, which are divided into channel A and channel B. Each group of samples is composed of sub-frames, that is, there are two sub-frames in one frame. The data length of the sub-frame is 32 bits, which contains the header code (Preamble), auxiliary data (Aux Data), audio data (Audio Data), as well as four-bit information and check codes. In the context of digital audio transmission, a sub-frame is 32 bits, equivalent to 4 bytes, and it is part of a frame that is 8 bytes. A block, composed of 192 frames, amounts to 1536 bytes, and each block can convey a total of 192 dual-channel samples.

The 0–3 bits Preamble is used to represent the beginning of a sub-frame. There are three forms, X, Y, and Z, which, respectively, indicate that the sub-frame is channel A, channel B, or the starting sub-frame of a block (channel A).

The 30-bit channel status bit (C) is the key bit for us to restructure the protocol. This bit is the same as the user bit. One bit is transmitted from each sample group, and finally, a 192-bit channel status information is formed for each of the two channels. This 192-bit channel state information is divided into two different structures: Professional and Consumer. It is determined by the first bit. When set to 1, it is in Professional mode, indicating that the channel transmission is linear PCM audio data. When set to 0, it is in Consumer mode, representing other data.

**Figure 6:**
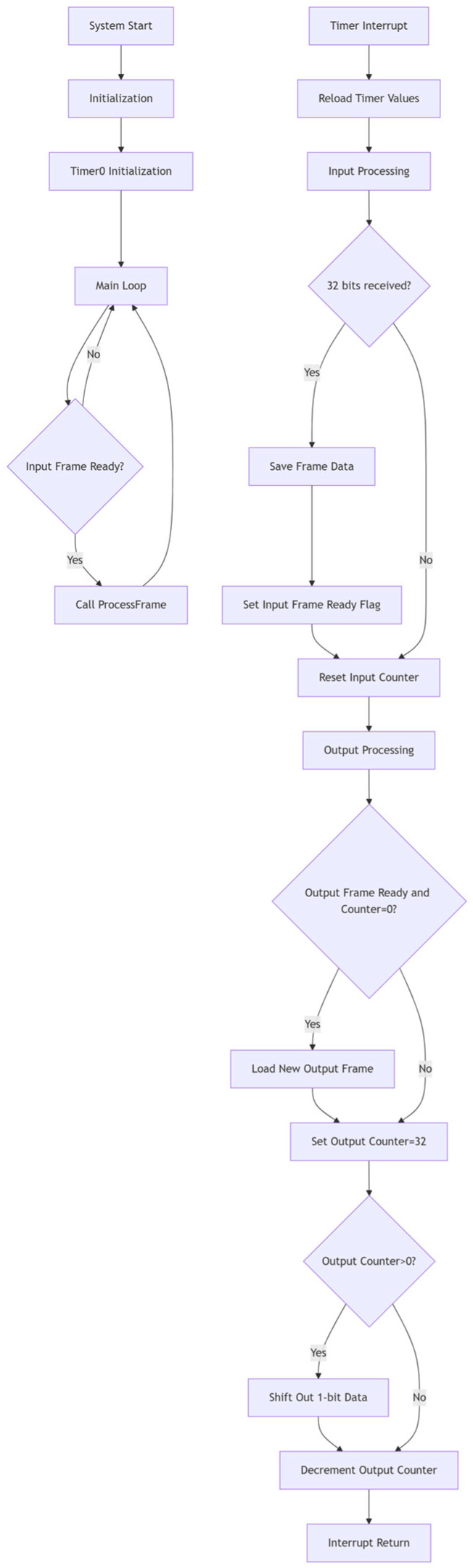
Main program flowchart (**left**) and timed interrupt service program flowchart (**right**).

Due to differing data types, in multi-service transmission scenarios, non-audio data are frequently detected via the channel state bit C in existing communication protocols designed for audio data and thus cannot be displayed. This study proposes a left–right channel transmission scheme that retains non-audio data. In the scheme, the CHS bit in the channel state data buffer control register is assigned a value based on the received header code. CHS = 0 indicates channel A, and CHS = 1 indicates channel B. The non-audio data is transmitted through channel A. At the receiving end, an OR operation is performed on CHS and C, and then the new value obtained from the operation is assigned to C. In order to achieve a lower hardware implementation cost of the system, a 51 single-chip microcomputer is adopted in the research to realize this function. The program adopts a structured design, including the main Program S1, the timer interrupt service program, and the frame processing (ProcessFrame) function. The flowcharts of the main program and the timer interrupt service program are shown in Figure 6.

One major difficulty in designing timer interrupt service programs is ensuring continuous data input and output. If the logic design follows the principle of sending one frame first after processing one frame after an interruption and then processing the next frame, there will be a short period of program execution time between each frame, which will cause the next stage to fail to receive correctly. Even if it can be received normally, only intermittent audio signals can be obtained at the final audio output end. Therefore, this study adopted a double-buffer design: using two buffers, out_buf0 and out_buf1, and a pointer, out_ready_flag, pointing to the buffer that is currently being output. The output shift register is always the value of the current output buffer because the output shift register is gradually shifted out during the output process. After a frame output is completed, the value of the current buffer is reloaded into the output shift register. If the backup buffer is not in the ready state, the value of the current buffer remains unchanged during the output process. However, when a switch is required, overwrite the output shift register with the value of the backup buffer. Through the above design, output frame preloading is achieved to ensure continuous output of 32-bit data without intervals.

**Figure 7:**
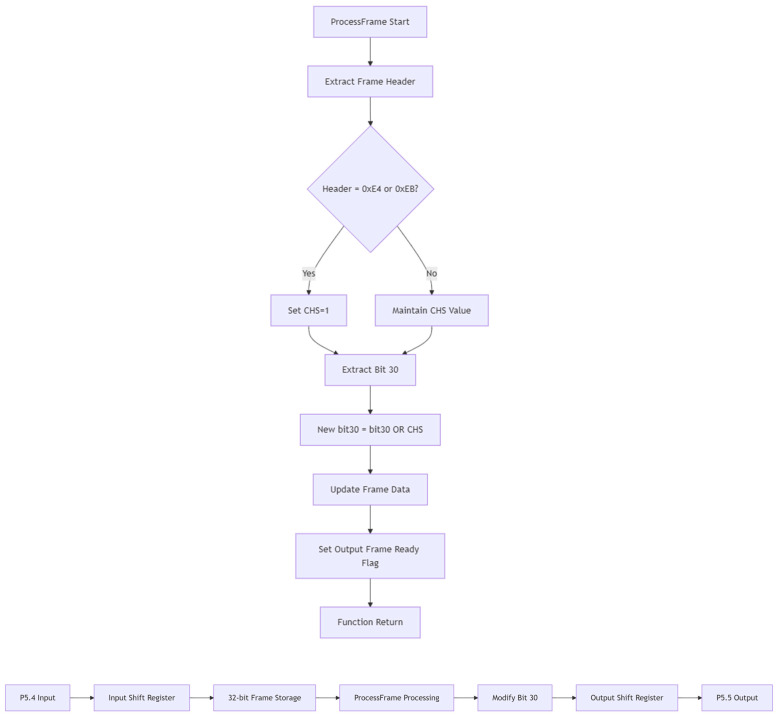
Frame processing function flowchart (**upper**) and Data Flow Diagram (**lower**).

The structure of the frame processing function is shown in Figure 7. The core of the function’s operation is to assign different values to the 30-bit channel state bit C based on different frame header states. There are three frame header states, X, Y, and Z, which, respectively, indicate that the frame channel is channel A, channel B, or the starting sub-frame of block A (also channel A). For the function, X and Z can be regarded as one category. Different operations on the A/B channels can be achieved by detecting the frame header Y. In IEC60958, the frame header does not follow the Biphase Mark Code (BMC) encoding rule, which is a phase modulation coding method that integrates clock and data signals into a single stream. Only the frame header will have three consecutive identical bits. This feature simplifies program design and eliminates the worry of interfering data from 8 to 31 bits. There are two sets of data in the frame header Y state, namely E4 and EB. When E4 and EB are detected for 8 consecutive bits, set CHS to 1; otherwise, set it to 0. Then, perform the AND operation between CHS and C to ensure that the channel state bit C of the non-audio data transmission channel B is always 1, while the channel state bit C of the audio transmission channel A remains unchanged.

The adjusted data transmission status is presented in Table 3. The data will not be transmitted normally, only when non-audio data appears in channel B. This not only guarantees the normal transmission of digital signals in channel A but also maintains the error detection function for audio transmission in channel B. If the traditional approach of using a single-chip microcomputer to read the clock at a certain stage for data transfer is adopted, it implies that each bit of non-audio data has to undergo additional register processing. This not only imposes higher requirements on register size and computing power but may also result in increased latency. The proposed scheme in the research naturally utilizes the characteristics of stereo transmission. It does not necessitate additional data processing. Only the channel state bits appearing once every 16 bits require reassignment, significantly simplifying the system structure and reducing the system’s implementation cost.

## 4. Design of Optical Transmitter and Receiver

**Figure 8:**
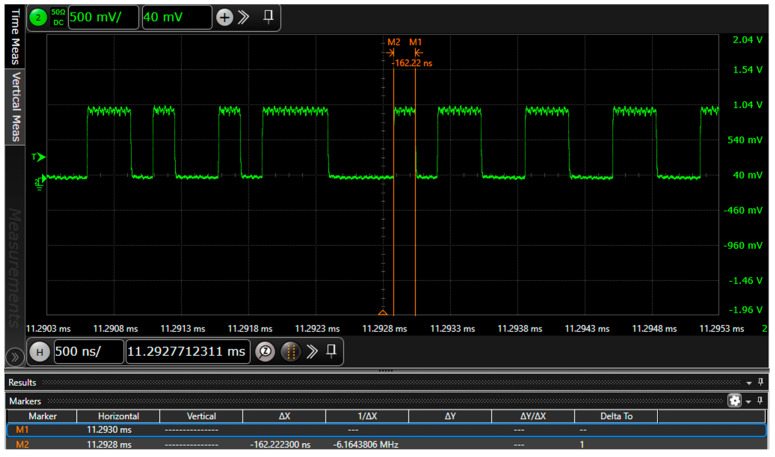
The transmission rate of AES3-encoded data modulated onto the LED.

Before designing the optical communication link, it is essential to determine the data rate to be transmitted to design a communication system that meets the performance requirements. The data transmission rate on SDIN/SDOUT in the I2S protocol is equal to the sampling frequency × bit width × number of channels, and numerically, it is equal to the frequency of the Bit Clock. The transmission rate remains unchanged after frame processing by the single-chip microcomputer. IEC60958 utilizes BMC for data transmission. The data rate doubles after being encoded by BMC. In the IEC60958 protocol, a basic frame consists of 36 bits, among which only 24 bits store sampling data. Thus, the data rate is increased to 1.5 times the rate of the previous step. The final AES3 data stream transmission rate modulated onto the LED is calculated as follows: transmission rate = sampling frequency (48 KHz) × bit width (24) × number of channels (2) × 2 × overhead factor (1.5) = 6.144 Mbps. The AES3 mixed-coded output data stream, as shown in Figure 8, was detected by an oscilloscope. Its data transmission rate is 6.16 Mbps, and the error between the two is small, which is in line with the theoretical calculation results.

**Figure 9:**
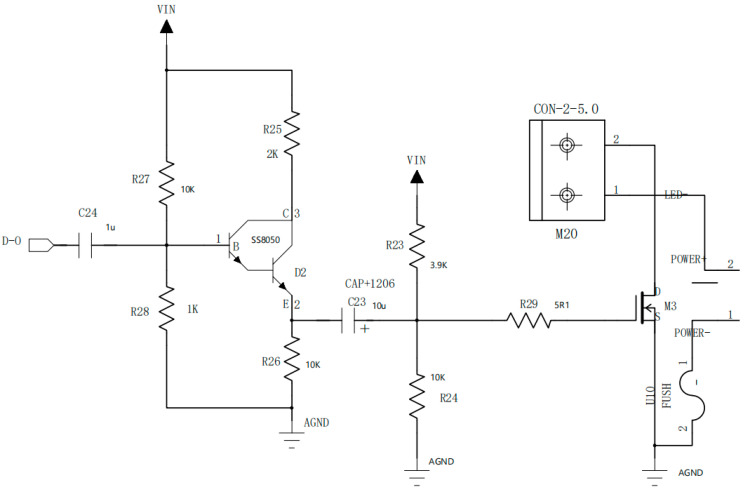
The circuit structure of the system’s optical transmitter.

The visible light link consists of an optical transmitter, a channel (free space), and an optical receiver. The structure of the optical emission module is shown in Figure 9. It adopts the OOK-NRZ modulation mode. The optical transmitter circuit consists of two cascaded stages: a first-stage transistor common-emitter amplifier and a second-stage MOSFET common-source amplifier. The AES3 signal from the encoder undergoes current amplification through the SS8050 transistor to boost power, enabling effective drive of subsequent MOSFET stages. The current amplification factor is calculated as follows:

(2) $$Ai=(1+\beta )*\left(Re/\left(Re+RL^{\prime }\right)\right)$$

Here, RL’ represents the AC load, typically defined as Re//RL (where Re and RL are connected in parallel), Re represents the emitter resistance, and RL represents the DC load.

The system employs the PD84001 MOSFET (STMicroelectronics, Shanghai, China), which operates in saturation mode during normal operation to achieve transconductance amplification. The drain current (I_D_) is controlled by variations in the gate-source voltage (V_GS_), with the relationship expressed as

(3) $$I_{D}=\frac{1}{2}\mu_{n}C_{ox}\frac{W}{L}{\left(V_{GS}-V_{TH}\right)}^{2}$$

Since port 1 connects to the LED’s anode and port 2 to its cathode, the MOSFET and LED are connected in series. Changes in drain current modulate LED brightness, thereby achieving signal modulation. According to the IV characteristic curve of PD84001, selecting the transconductance maximum operating point enhances the sensitivity of the LED current to signal amplitude variations, effectively improving modulation depth. Low-pass filtering is implemented using C24 and C23, while bias resistors R23, R24, R27, and R28 ensure optimal voltage distribution for SS8050 (Jiangsu Changjing Electronics Technology, Beijing, China) and PD84001. The R29 resistor suppresses signal reflections.

**Figure 10:**
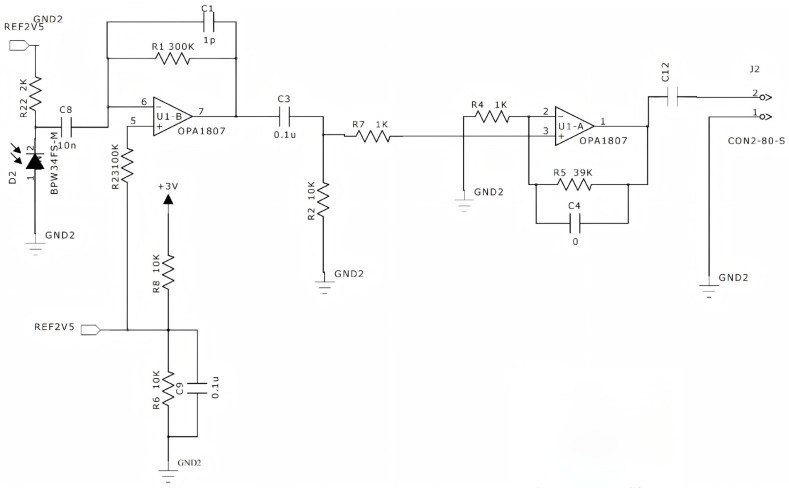
The circuit structure of the system’s optical receiver.

The optical receiver circuit is composed of two amplification stages, as shown in the lower part of Figure 10: a transimpedance amplifier (LT1807-based) and an inverting amplifier (also LT1807-based). The LT1807 (Analog Devices, Beijing, China) is a single/dual-channel low-noise rail-to-rail input/output unit gain stable operational amplifier, featuring a gain bandwidth product of 225 MHz, a conversion rate of 140 V/μs, and an output current of 85 mA. The transimpedance stage converts the weak current signal from the photodiode (PD) into a measurable voltage signal, enhancing signal amplitude and sensitivity for subsequent processing. R1 acts as the feedback resistor, while C1 serves as the feedback capacitor, with R1’s value determining the first stage’s gain. The LT1807’s positive input terminal supplies a 2.5 V reference voltage to provide the DC component. The inverting amplifier stage inverts and amplifies the input signal, where the amplification factor is determined by the ratio of R5 to R4. The closed-loop voltage gain is calculated as follows:

(4) $$Gain(Av)=\frac{V_{\mathrm{out}}}{V_{\mathrm{in}}}=-\frac{R_{f}}{R_{\mathrm{in}}}$$

Here, R_f_ = R5 and R_in_ = R4.

The amplified signals are transmitted through ports 1 and 2 to the decoder for decoding.

**Figure 11:**
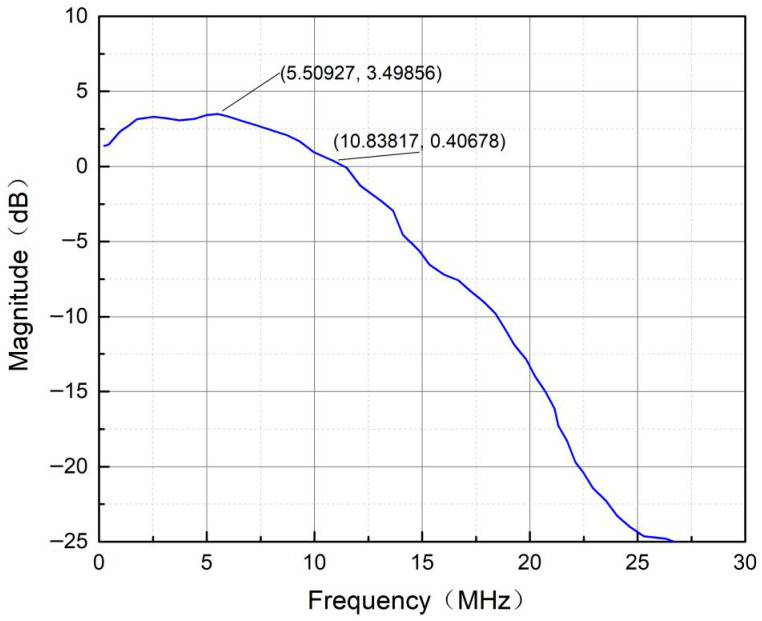
The measured overall system bandwidth (including the LED transmitting circuit and receiver circuit) amplitude–frequency response curve of the visible light communication link.

Under the daily lighting conditions in the laboratory (116 lx) and a communication distance of 3.2 m, the −3 dB bandwidth of the VLC system composed of the visible light transmitting end and the receiving end was approximately 10.8 MHz, as tested by a network analyzer (Figure 11). Shannon’s theorem states that in a communication system, the maximum achievable transmission rate (data rate) depends on the bandwidth and signal-to-noise ratio (SNR) and provides a theoretical limit calculation formula:

(5) $$C=B{log}_{2}(1+SNR)$$

Here, C represents the channel capacity (unit: bps, bits per second), B is the bandwidth (unit: Hz), and SNR is the signal-to-noise ratio (dimensionless). By substituting the data rate to be transmitted and the system bandwidth, it can be theoretically concluded that communication requirements can be met as long as the SNR exceeds −3.4 dB. This value is relatively low. In a typical indoor VLC environment, without shading and without adding lenses, the SNR is usually within the range of 10–20 dB. By adding shading treatment and optical filters, the SNR can reach 25–40 dB. Meanwhile, considering the requirement of the bit error rate (BER), with 10^−6^ as the critical value for accurate transmission, according to the BER calculation formula in the Additive White Gaussian Noise (AWGN) channel under the OOK-NRZ modulation mode [14]

(6) $$BER=Q(\sqrt{SNR})$$

For an OOK-NRZ signal operating at 6.1 Mbps with a bit error rate (BER) exceeding 10^−6^, the theoretical signal-to-noise ratio (SNR) should be no less than 13.5 dB. In indoor environments where both transmitters and receivers are equipped with optical lenses, the actual SNR significantly exceeds the theoretical minimum of 13.5 dB.

## 5. Test Results and Analysis

### 5.1. System Performance Analysis

**Figure 12:**
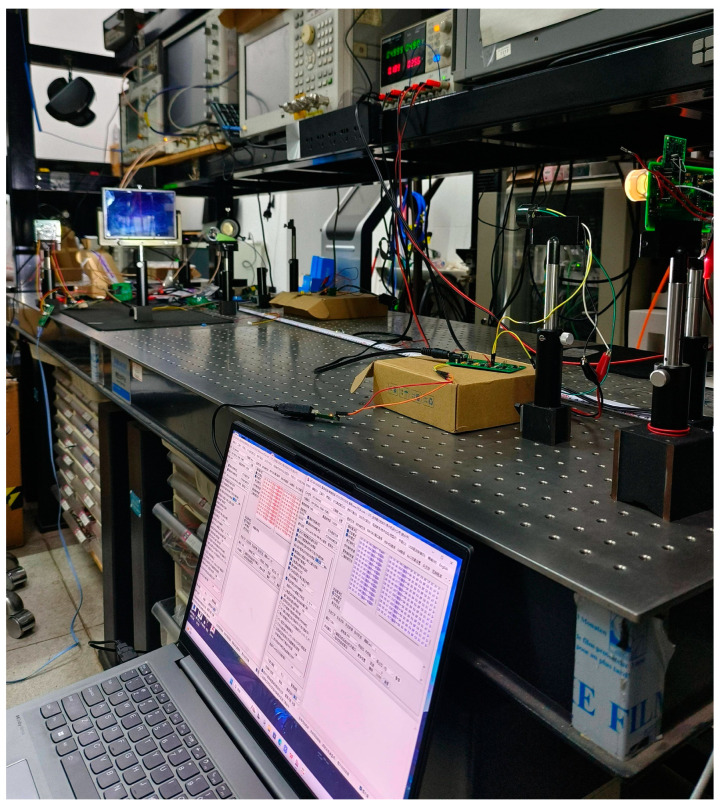
Test of visible light speech character simultaneous transmission system.

Based on the theoretical investigation and computational analysis of the aforementioned visible light multiplexing system, we built a VLC system on a single-layer PCB board (8 cm × 5 cm) that can simultaneously interpret voice and characters, as shown in Figure 12. The system consists of two identical hardware circuit boards. According to the different configurations of the internal single-chip microcomputer registers, they are divided into the transmitter and the receiver. The audio processing chip of the system is MAX9860 (Maxim Integrated Products, Shanghai, China), and the single-chip microcomputer selected is the 1T high-speed 51 single-chip microcomputer STC15W408s (STCmicro, Beijing, China), which supports a maximum main frequency of 35 MHz and actually adopts a main frequency of 24 MHz, providing a shorter instruction cycle and enabling the reconfiguration of the shift register to be completed within 1 us. The system uses a microphone to collect external voice signals, which are provided by the mobile phone playing the recorded audio files. Program burning and serial port control were carried out using the downloader STC-USB Link1D and the supporting software stc-isp-v6.92k, and character data were output to the system through the simulated serial port of the computer. The audio signal output at the receiving end of the system is collected by the oscilloscope, and the character data output is collected and converted by the software stc-isp-v6.92k. The system was powered by 5 V, and the maximum communication distance was tested by placing a plane mirror. The LED operates at 5.4 V with a current of 0.36 A.

**Figure 13:**
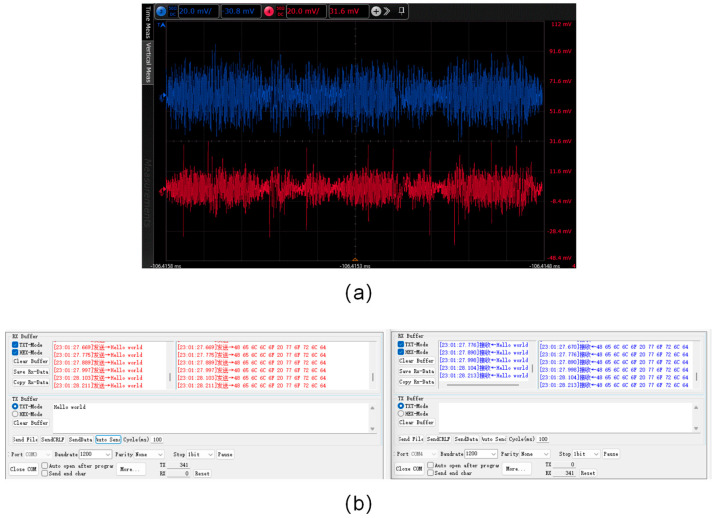
System test results: (**a**) input (blue) and output (red) audio signal comparison; (**b**) input (left, red) and output (right, blue) character data comparison.

Figure 13 illustrates the communication performance of the visible light-based voice and character simultaneous interpretation system. Figure 13a presents a comparative analysis of the input and output audio signal waveforms recorded via the oscilloscope. The blue waveform represents the audio signal captured by the microphone, whereas the red waveform denotes the audio signal acquired by the headphones. The waveforms of the two signals exhibit high similarity. Figure 13b displays the input and output character data recorded through the serial port software (stc-isp-v6.92k). The text displayed in red on the left represents the input character sequence, “Institute of Semiconductors, Chinese Academy of Sciences”, while the text in blue on the right corresponds to the received character sequence. A total of 341 characters were successfully transmitted, received, and accurately displayed. The output audio signal closely matches the input audio signal, enabling clear and precise reproduction of the electronic sound emanating from the headphones. Meanwhile, the character sequences transmitted via the serial port are identical to those received, achieving the expected service multiplexing function.

**Figure 14:**
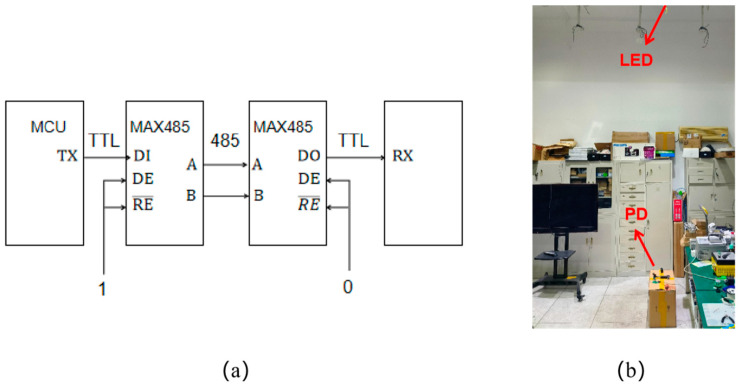
(**a**) TTL level to 485 circuit (**b**) broadcast communication test.

By incorporating an additional 485-level conversion circuit, the system can be seamlessly integrated into the existing power network, enabling highly secure broadcasting communication in confined spaces like aircraft and space capsules. As depicted in Figure 14, the transmitting end of the system is connected to the ceiling light in the laboratory, and the receiving end can still restore clear, error-free voice signals. The acceptance range is determined by the scattering angle of the light source at the transmitter.

The robustness of the system is discussed next. First, the system’s signal-to-noise ratio is calculated to verify the assumptions made in Section 4. In this visible light multiplexing system, the main sources of noise include two parts: the circuit noise of the receiver itself and the ambient light noise. The output signal of the receiver was obtained by using an oscilloscope. The noise power was obtained by subtracting the original clean signal from the output signal. According to the SNR calculation formula,

(7) $$SNR=10lg\frac{P_{s}}{P_{n}}$$

The actual power calculated is approximately 14.2 dB. The measured data rate of 6.16 Mbps and system bandwidth of 10.8 MHz yield a theoretical minimum SNR requirement of −3.4 dB according to Equation (4). Our system, operating in a typical indoor environment, maintains an SNR of 17.6 dB well above this threshold (and above the 13.5 dB required for a BER < 10^−6^ with OOK-NRZ), confirming the robustness of the link design.

We investigated the impact of illumination on system stability through two key tests: maintaining operational LED illumination levels and evaluating ambient light effects. In a dark, enclosed space without external lighting, the system demonstrated reliable duplex communication at 170–400 lx illumination levels with a 5 m transmitter–receiver distance. Reducing the distance further lowered the minimum illumination threshold required for normal operation. The 330 lx upper limit was implemented during experiments to protect LEDs from burnout, as excessive LED intensity could cause photodiode (PD) saturation and compromise communication. Our tests indicate that the system operates effectively in general indoor environments with broader illumination ranges. To assess ambient light interference, we conducted experiments with the receiver operating at 43 lx, 81 lx, and 134 lx illumination levels while the system remained powered off, maintaining LED illumination at approximately 350 lx. All three conditions ensured stable system performance, with clear voice transmission quality and error-free character data reception. Therefore, through the analysis of test results, the visible light communication multiplexing system designed in this study has strong anti-interference capability and is very compatible with the existing indoor lighting network.

To comprehensively evaluate the performance and positioning of our proposed system, a comparative analysis with other recently reported VLC systems is presented in Table 4. The comparison encompasses key metrics such as data rate, transmission distance, supported services, multiplexing technique, and core innovation.

Our system distinguishes itself by its unique application focus and design philosophy. Instead of pursuing the ultimate data rates, multi-user capacity, or complex physical layer signal processing, we address the practical and often overlooked need for low-cost, concurrent transmission of heterogeneous services (audio and data) for a single user over a single optical channel. Our achieved data rate of 6.16 Mbps and transmission distance of 3.2 m are sufficient and appropriate for target applications like clear voice communication and sensor data/text transmission in indoor environments (e.g., hospitals and aircraft cabins). The key advantage lies in the system’s simplicity and cost-effectiveness, achieved through the innovative reconfiguration of the standardized I2S/AES3 protocol. This approach allows the use of low-cost microcontrollers and commercial white LEDs, avoiding the need for expensive light sources, complex DSP/FPGA, or active optical components required by many other high-performance systems.

Based on the test results, the heterogeneous service simultaneous transmission architecture system designed in this research, which utilizes VLC, has largely achieved the function of mixed transmission for different services. Under stable transmission conditions, the system can support a maximum transmission distance of 3.2 m, which is suitable for indoor applications, and can communicate effectively under natural lighting conditions. The system supports audio signal transmission within the range of 1 kHz to 21.6 kHz, as well as serial port signal transmission at a rate of 300–1200 bits and can easily be integrated into the existing lighting network. This system provides a solution for the wireless communication multiplexing requirements in RF-sensitive areas, filling the gap that the optical wireless fusion multiplexing system is difficult to apply in RF-sensitive areas and is conducive to achieving full coverage of service multiplexing.

### 5.2. Discussion on System Limitations and Bottlenecks

Notwithstanding the successful demonstration of multi-service multiplexing, the proposed system’s performance is bounded by several inherent constraints of its minimalist design.

Data Rate Ceiling: The aggregate data rate is fundamentally constrained by the modulation bandwidth of the commercial white LED, the maximum master clock frequency of the low-cost microcontroller (MCU), and the fixed overhead of the BMC encoding protocol. These factors collectively set a practical upper limit for the current architecture.

Microcontroller Processing Capability: The chosen STC15W408s MCU provides cost-effectiveness at the expense of processing headroom. Its 24 MHz operating frequency is fully utilized for protocol execution, leaving minimal resources for implementing more advanced, computation-intensive signal processing tasks such as digital equalization.

Channel Dependent Performance: The system’s robustness is primarily validated in line-of-sight (LoS) conditions. Performance in non-line-of-sight (NLoS) scenarios is susceptible to multipath distortion and inter-symbol interference, as the simple OOK-NRZ modulation and receiver lack inherent mechanisms to mitigate these effects.

## 6. Conclusions

This study designed and implemented a multi-service multiplexing system based on VLC, which successfully achieved hybrid transmission of audio signals (1 kHz–21.6 kHz) and character data (300–1200 bps) over a single optical channel. Through the integration of dynamic level control and the utilization of the channel state bit redefinition function, the system overcomes the limitations of the traditional pure-audio optical communication system and enables the synchronous transmission of heterogeneous data streams.

The experimental results indicate that the developed system is capable of consistently recovering voice and character data across a communication range of up to 3.2 m within standard indoor lighting conditions. The system features a compact hardware design, low implementation cost, and strong scalability, making it suitable for deployment in RF-sensitive environments such as aircraft cabins, hospitals, and industrial control rooms.

This work not only provides a practical solution for multi-service optical wireless communication in restricted scenarios but also offers a reference for the integration of VLC with existing wired and wireless infrastructures. Future research will focus on enhancing data rates and transmission distances by employing advanced optoelectronic components like micro-LEDs and modulation schemes. Further integration with IoT platforms and the development of adaptive algorithms to improve robustness in dynamic environments are also key directions. Finally, the integration of a hybrid communication system with the existing RF network is also a promising path.

## Figures and Tables

**Table 1:** Comparison of multiplexing techniques for VLC.

Multiplexing Technique	Key Challenges in VLC	Advantages of Time Division Multiplexing (TDM) in This Context
Frequency Division Multiplexing (FDM)	LED nonlinearity and bandwidth limitations	Avoids frequency-domain processing; simpler transceiver design
Code Division Multiplexing (CDM)	Complex code synchronization	No complex spreading codes; simpler time-slot management
Wavelength Division Multiplexing (WDM)	Costly multi-wavelength sources	Uses a single white LED, low cost, and simple implementation

**Table 2:** Key parameters of the TDM transmission scheme.

Parameter	Values
Master Clock Frequency	12.288 MHz
Sampling Rate (fs)	48 kHz
Word Clock Frequency	48 kHz
Bit Clock Frequency	2.304 MHz *
Audio Bit Depth per Channel	24 bit

* note: BCLK = 2 × 24 × fs (for 24-bit sub-frames per channel).

**Table 3:** The updated status of non-audio and audio data transmission for channel A and channel B after protocol reconfiguration. “C” represents the original channel state bit, and the value of “CHS” depends on whether the channel is channel A or Channel B. “C*” represents the new channel state bit, and its value is equal to the value after the and operation of “C” and “CHS”.

Data Type	C	CHS	C’=C&CHS	Data Mode
Non-Audio Data	0	1 (A channel)	1	Normal
	0	0 (B channel)	0	Blank
Audio Data	1	1 (B channel)	1	Normal
	1	0 (A channel)	1	Normal

**Table 4:** Performance comparison of the proposed system with other VLC systems.

Reference/System	Data Rate/Bandwidth	Transmission Distance	Services Supported	Cost and Complexity	Core Innovation/Focus
Gunawan et al. [6]	21.01 Gbit/s	2–4 m	Data streams (2 per wavelength)	High software complexity, relies on a centralized controller and iterative optimization algorithms.	Ultra-high capacity; multi-user support via advanced modulation and beam steering.
Chen et al. [7]	750 Mbps	1.3 m	Data streams	Specialized modulation schemes and cooperative control algorithms; high implementation complexity.	High user capacity and low latency for bidirectional network access.
Jia et al. [4]	41.8 MHz	0.6 m	Data streams (O-OFDM)	High hardware cost (FPGA); overly complex for simple point-to-point communication.	BER performance improvement and PAPR reduction in multipath channels using pre-coding.
Chen et al. [5]	500 Mbps	2.15 m	Data streams (NOMA)	Hybrid design requiring FPGA development; high complexity in hardware design and debugging.	Maximizing system sum rate and user QoS under non-uniform user distribution.
Tang et al. [8]	10 Mbps	2.8 m	IoT data; Energy Harvesting	Flexibility and need for rapid prototyping, potentially relying on specific hardware (e.g., USRP).	Sustainable IoT operation via SLIPT and optimized resource allocation.
This work	6.16 Mbps	3.2 m	Audio + Character Data	Very low hardware and software costs (single-layer PCB and microcontroller).	Low-cost, single-channel multi-service multiplexing for a single user using a reconfigured audio protocol.

## Data Availability

The data of this study are available from the corresponding author upon request.

## Supplementary Materials

The following supporting information can be downloaded at https://www.mdpi.com/article/10.3390/s25237207/s1, Program S1: audio_processor.c.

## Abbreviations

The following abbreviations are used in this manuscript:

IoT	Internet of Things
VLC	Visible Light Communication
RFID	Radio Frequency Identification
ICUs	intensive care units
TDM	Time-Division Multiplexing
ADC	Analog to Digital Converter
DAC	Digital to Analog Converter
AGC	Automatic Gain Control
WCLK	Word Clock
BCLK	Bit Clock
PGA	Programmable Gain Amplifier
C	Channel Status Bit
BMC	Biphase Mark Code
AWGN	Additive White Gaussian Noise
OOK	On-Off Keying
NRZ	Not Return to Zero
